# The Microscope

**Published:** 1874-03

**Authors:** Geo. B. Harriman

**Affiliations:** Boston, Mass.


### THE MICROSCOPE.

BY GEO. B. HARRIMAN, D. D. S., M. D., BOSTON, MASS.

The microscope was invented in the interest of science, and no individual can become familiar with physiological and histological new formations without thorough preparation, careful study and patient investigation.

A beginner in microscopic manipulation will find it hard work in the higher branches of natural science, unless he is well versed in practical physiology and chemistry, to become a master in this specialty.

Spherical aberration is known by the want of good definition, when the object is blurred and indistinct. When the objective is not properly corrected for chromatic aberration, colored lines will be seen—reddish if over-corrected, blue if not corrected enough. A first-class object-glass should not show colored fringes around the object and should be perfectly achromatic ; the higher the angle of aperture, the more valuable is the objective.

Nearly every gentleman who has a microscope has had the question asked him, "How much will your microscope magnify ?" My answer is : it usually depends upon the power of the objective and the eye-piece used. If every object-glass was marked by its maker accurately, and every eye-piece correct, there would be no difficulty of at once determining its exact magnifying power. But some makers are not at all particular in making their objectives.

Theoretically, a 1-75 object-glass will magnify 750 diameters ; a 1-50, 500 and a 1-12, 125 diameters, at ten inches distance from the center of the objective. The eye-piece also magnifies the object ; the higher the power of the eye-piece, the more is the object increased in size. The highest eye-piece that Mr. Tolles makes is the 1-8, which magnifies 80 diameters ; 80 times 750 is 60,000. With a 1-75 objective and 1-4 eye-piece, the magnifying power would be 30,000 diameters, or forty times seven hundred and fifty. But here we meet with much difficulty — the want of light. The higher the magnifying power of the eye-piece, the less light we obtain to view the object, and have to resort to achromatic condensers, which are made for the purpose of condensing the light upon the object.

The 1-4 in. object-glass makes a very good condenser. The student should always commence with low powers, one inch and 1-4 in. object-glasses, and continue to increase their objectives until they are able to use the highest powers that are produced. For examining object-glasses, I would recommend J. D. Moller's Diatomaceen Probe-Platte, which contains twenty-one nicely mounted test objects, from the coarsest Eupodiscus Argus to the finest Amphipleura pellucida.

The 1-75 * and 1-50 † immersion objectives made for me by Mr. Robt. B. Tolles, of this city, are indeed most wonderful productions—the definition and penetrating power of these two objectives cannot be excelled ; the magnifying power, with medium high power eye-piece, is from ten to fifteen thousand diameters. The light is good.

The *Boston Journal of Chemistry*, for Sept., 1873, says :

"Boston stands pre-eminent in the production of exquisite and wonderful optical instruments. Mr. Tolles has just achieved the great result of producing a 1-75 objective for microscopic use, a glass of such difficult construction that we believe no optician has ever attempted it before.

"The power of this objective is such that a *single white blood corpuscle covers the entire field of vision.*

"Mr. Tolles has produced two of the finest 1-50 objectives ever constructed, one of which is in this city ; the other is in the hands of a western gentleman (G. W. Morehouse, Wayland, N. Y.). The angular aperture of one is 120°; that of the other, and the last constructed, is 165°.

"These objectives are of great excellence and, in the opinion of competent microscopists, far surpass in defining power and clearness of field those of European make."

---

* Cost of 1-75, ($400) four hundred dollars.

† Cost of 1-50, ($225) two hundred and twenty-five dollars.

